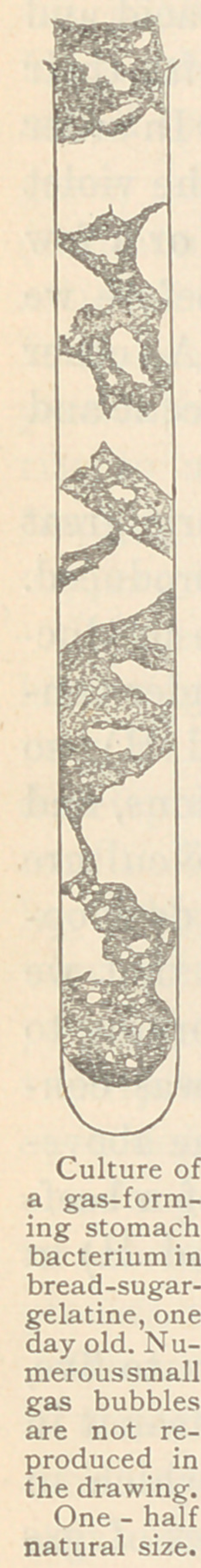# On Certain Fermentative Processes in the Alimentary Canal and the Micro-Organisms by Which They Are Produced

**Published:** 1886-03

**Authors:** 

**Affiliations:** Berlin


### Independent Practitioner.

Vol. VII. March, 1886. No. 3.

### Original Communications.

### ON CERTAIN FERMENTATIVE PROCESSES IN THE ALIMENTARY CANAL AND THE MICRO-ORGANISMS BY WHICH THEY ARE PRODUCED.

BY PROF. DR. MILLER, BERLIN.

(Continued from the February number.)

Some further questions of interest in this connection are the following: At what stage in the process of digestion, or at what percentage of acid in the contents of the stomach does fermentation cease? How much of a given antiseptic—hydrochloric acid, salicylic acid, etc., must be given in order to bring about a cessation of an abnormal fermentation in the stomach? I attempted to effect a solution of this question through the following experiments:

I chewed up a portion of meat and bread and added a small quantity of sugar with sufficient milk to make a thick paste. I then infected this mixture richly with three kinds of stomach fungi which are characterized by the large quantities of gas that they produce, and added to it an equal quantity of four per cent. peptone-sugar-gelatine. After it had stood for an hour in the incubator, it was divided into a number of portions of 20 cc. each. To each of these portions I added hydrochloric acid in increasing quantities, so that the first received four parts of H. cl. to 10,000, and the last two to 1,000. After this they were poured into test tubes, and the level of the mixture in each tube accurately marked. The portions being kept at 20° C. soon solidified, and after eight to twelve hours, in all the tubes where fermentation took place an elevation of the surface of the gelatine was observed, due to the formation of bubbles of gas, and in those tubes containing the smallest quantity of H. cl. the mixture was in part driven quite out of the tube. I found that not till the proportion of H. cl. reached 1.6 to 1,000 did the surface of the mixture remain at a constant level, or in other words, did the fermentation cease. Salicylic acid effected the same in the proportion of 0.4 to 1,000. If, therefore, in a food mixture we wish to prevent fermentation, or to arrest it when once begun, we must add 1.6 grams H. cl., or 0.4 grams salicylic acid to each 1,000 grams of the mixture.

In the human stomach, however, we have to deal, not with a total lack of H. cl., but rather with a diminution in the quantity of the same, so that we require only to supplement the acid already present. When digestion is at its most active stage, the normal proportion of H. cl. in the stomach, at which all fermentations under normal conditions disappear, is 2 to 1,000, and since fermentation does not take place till the proportion of H. cl. has been reduced to 1.6 to 1,000, we must, in cases of continued fermentation in the stomach, expect a lack of H. cl. equal to at least 0.4 grams per liter of stomach contents, and this quantity must be administered in order to restore the normal condition.

According to the determinations of Bidder and Schmidt, ten to twenty liters of gastric juice are secreted daily. If we consider this estimate as two to four times too high, and take five liters as the real quantity, then we have still ten grams H. cl. (forty grams H. cl. solution of sp. gr. 1,1233) which are daily poured into the human stomach. Under such circumstances we can hardly expect to produce much impression by the three to ten drop-doses recommended in text-books. The quantity of any given antiseptic which is necessary to suspend fermentation in the human stomach depends upon the intensity of the fermentation and the quantity of the stomach contents; in any case we must calculate upon considerable and repeated doses of H. cl. if we wish to produce a marked impression. In case of the ordinary lactic-acid fermentation, outside of the stomach the process will, in a few hours, be retarded by the antiseptic action of the acid which is produced. In the stomach this action loses its signification, from the fact that the acid produced is speedily absorbed, and the contents of the stomach are a number of times daily replaced by neutral material. These experiments also show how small a change in the quantity or quality of the gastric juice is necessary to render a permanent fermentation (*dyspepsia chronica*) in the human stomach possible.

The objection may be urged against these experiments that, in the solidified condition of the mixture, the H. cl. could not have its full effect. The control experiments, however, made at the temperature of the human body (at which the mixture of course became liquid), confirmed the accuracy of the previous results. It may be further said that a portion of the H. cl. disappeared in combination with bases in the mixture, but I am satisfied from experiments not here described, that the loss from this source must have been very small, and was replaced by the acid present in the mixture at the beginning of the experiment, the reaction in each case being clearly acid.

I next attempted to determine the action of these fungi on carbohydrates, particularly their acid-producing power, being anxious to find the source of the acids of the human mouth. For this purpose I cultivated them in beef-extract-sugar solutions, in peptone-sugar solutions and in milk. Sixteen of the mouth fungi produced an acid reaction, four an alkaline, and five gave inconstant results. The corresponding numbers for the stomach fungi were nine, two and two; for the fungi of the intestines, six, five and three. The proportion of the acid-forming fungi in the mouth and stomach is, according to these results, much greater than in the intestines. Whether this condition is constant or not, could be determined only by a large number of series of experiments. The cultures in sterilized milk gave results slightly different from the above. It was, furthermore, not possible to draw a sharp line between those fungi which produced an acid, and those which produced an alkaline reaction, since in some cases the reaction was only slight and changed with time, while in other cases it was altered by a change in the amount of sugar present. One bacillus, which I tested in reference to this question, cultivated in three per cent. beef-extract solution, left the reaction neutral when one-tenth per cent. sugar was present; if the amount of sugar was increased, the reaction became acid; if it was diminished, the reaction became alkaline.

It is equally difficult, I think, to draw a sharp line between putrefactive or fermentative organisms, since many ferment organisms are capable of decomposing albuminous substances with development of putrefactive products, while on the other hand many organisms which pass as putrefactive, when brought into saccharine solutions give rise to fermentation without the production of a trace of the characteristic products of putrefaction. One of the mouth-fungi which I examined in reference to this question, readily dissolved coagulated albumen with development of bad smelling gases, among which $SH_2$ (Sulphuretted hydrogen) and $NH_3$ (Ammonia) could be easily detected. It also showed an inverting action, in that it converted cane sugar into Dextrose and Levulose. In the third place it split fermentable sugars into lactic acid, with production of $CO_2$ (Carbon dioxide ) In the fourth place it gave rise to an acid reaction in a solution of starch, while at the same time the solution acquired the capacity of reducing the oxide of copper; in other words, this fungus showed also a diastatic action.

In these different fermentations there undoubtedly arise in the later stages various secondary products, so that the changes which may be brought about by this one fungus, and the products developed under its action, make up a very large number. This result affords an explanation of the fact that in an open decomposing substance so many different products may appear, and renders very doubtful the supposition that, for every new product in the process of decomposition, a new organism must be present. The twenty to thirty different compounds which may be produced in an open decomposing solution, are in all probability not produced by one fungus alone. It is, however, not in accordance with the facts to assume that for every product, or indeed for every stage in the process, a new fungus must be introduced.

On account of the large number of the fungi under consideration, it was not possible to undertake a determination of the acid in each case. Formerly it was believed that there was a certain specific organism, which alone could give rise to lactic acid fermentation. In 1883, however, I showed that there were a number of fungi in the human mouth which possess the capacity of forming lactic acid from sugar, and to-day it is perfectly well established that this action, as well as the inverting or peptonizing, is very widespread among the Schizomycetes. This fact furnishes an easy explanation of the constant appearance of lactic acid in the stomach and intestines.

The determination of the acid was made in part by analysis, in part by crystallization of the zinc salt, and in part by the color test, recommended by Prof. Ewald.* By this simple test the detection of lactic acid is rendered very easy. Unfortunately, the test can be applied only under certain conditions, since not only lactic acid and its salts, but also tartaric, citric, malic and oxalic acid with their salts, as far as I have tested them, give the same reaction. In order to produce the yellow color, it is only necessary to add to the violet solution a piece of fruit (apple, grape, plum, tomato, etc.,) or a few drops of white wine. All these acids must be eliminated before we can consider the reaction a conclusive test for lactic acid. As other products of fermentation, I was able to determine formic, acetic and butyric acid, the latter, however, only in small quantities.

Macroscopically, these different fermentations show very great differences, particularly in respect to the amount of gas produced. Very often lactic acid fermentation takes place without the production of a trace of gas, whereas some of the fungi now under consideration produce very large quantities of gas ($CO_2$ and H), so that the gelatine in the culture tubes is torn in all directions, and frequently a part of it is driven quite out of the tube, or the culture vessel is ruptured by the presure of the gas. This copious development of gas can only be accounted for on the supposition that we do not have before us a pure lactic acid fermentation (according to the formula $C_6H_{12}O_6=2\ C_3H_6O_3$), a supposition which was confirmed by the analysis which showed the presence of the above-mentioned acids. During the fermentation of half a liter of a beef-extract-sugar solution I collected 250 cc. of gas ($CO_2$ and H) in three hours.

Of these gas forming fungi I would like to call attention to five, which regularly form large gas bubbles in the gelatine or tear it in pieces, as represented in the figure. One of these fungi, which in albuminous substances also produces considerable quantities of gas ($SH_2$ and $NH_3$) was found in the fæces as well as in a gangrenous tooth pulp. Its presence in the latter place explains the manner in which dental abscesses may often be formed. If a tooth with a necrosed pulp is filled without previously removing the pulp and sterilizing and filling the root canal, the gas which is formed may make its way through the foramen at the end of the root, or drive particles of the decomposing pulp through with it. The irritation

* One drop chloride of iron, two drops carbolic acid, ten cc. water—violet color, which becomes yellow on adding lactic acid.

thereby produced gives rise (in most cases immediately) to an inflammation of the pericementum. In such cases it was formerly a custom, still practiced by a few, to drill a vent in the root to allow the gases and other products of decomposition to escape. It is not difficult to find among the clients of many German dentists mouths with a number of these vent-holes, which constantly discharge offensive pus, gas, etc., into the oral cavity.

Culture of a gas-forming stomach bacterium in bread-sugar-gelatine, one day old. Numerous small gas bubbles are not reproduced in the drawing. One-half natural size.

Three of the other four gas-forming fungi I found in the stomach, and one in the fæces. It is not difficult to see the distubances that might be produced by an abnormal development of these fungi in the stomach or intestines. A further question of interest regards the peptonizing action, particularly of the fungi of the intestines. By far the greater number of the different species which I have examined grow well on boiled white of egg, and can therefore be said to possess a peptonizing action. In some cases the albumen became completely liquefied by the action of the fungi. Whether the fungi form more peptone than they need for their own nourishment, and whether and to what extent they may thereby be of use to the human body, is a question whose solution presents difficulties not yet overcome. In a large quantity of sterilized white of egg infected with a comma bacillus, I found traces of peptone at the end of the third day.

Very few of the fungi here treated of were found to possess any marked diastatic action. Of nine species which I examined specially with reference to this property, only one gave a decidedly affirmative result. By the action of this fungus starch was converted into sugar, and this again into acid.

It is generally taken for granted that a bacterium which grows on boiled potato must possess a diastatic action. Such evidence is, however, of little value, because the fungus is not dependent upon the starch of the potato alone, but may derive sufficient nourishment from the sugar and albumen of the potato to maintain its existence for some time.

Not one, out of more than fifty species that I examined, belonged to that group of micro-organisms called anærobian; *i. e.* no one of them grew better or exclusively under exclusion of atmospheric air. On the other hand, I found every gradation, from those which showed no development whatever under exclusion of atmospheric air, to those which grew equally well with or without it.

The following conclusions may be drawn from the experiments described above:

(1.) A large number of the fungi of the alimentary canal are not restricted to one portion of it alone, but may develop either in the mouth, stomach or intestines.

(2.) In by far the greater number of cases the gastric juice will not prevent the entrance of fungi into the intestines. All fungi which I have examined may pass the stomach without losing the power of development, provided they are swallowed at the beginning of a meal. If, on the other hand, digestion is at its most active stage (two to three hours after the beginning of a meal), then those fungi more sensitive to the action of acids will be destroyed before they reach the intestines.

(3.) Lactic acid fermentation may continue in the stomach until the percentage of H. cl. reaches 1.6 to 1,000. If too little H. cl. is secreted, or too much food taken at once, the fermentation may become permanent. Diseases of the stomach, general disorders of health, fever, etc., accelerate the fermentation by interfering with the normal secretion of gastric juice.

(4. Fermentation in the stomach may be more readily arrested with salicylic than with hydrochloric acid.

(5.) A large number of the fungi of the alimentary canal cause lactic acid fermentation in solutions of carbo-hydrates, whereby the frequent appearance of lactic acid may be accounted for. Other ferment acids, acetic, butyric, etc., I have observed less frequently and in smaller quantities.

(6.) Five of the species examined caused fermentation with formation of large quantities of gas, chiefly $CO_2$ and H.

(7.) It is impossible to make an exact division between those fungi which produce an acid and those which produce an alkaline reaction in a given solution; also between ferment and putrefactive fungi.

(8.) The majority of the fungi which I have examined manifested a peptonizing, very few a diastatic action.

(CONCLUDED IN THE NEXT NUMBER.)

## Figures and Tables

**Figure f1:**